# Connections associated with a healthy spirituality: are these unrecognized intermediary determinants that shape health inequities in Canadian young people?

**DOI:** 10.1186/s12889-023-16060-5

**Published:** 2023-06-17

**Authors:** Valerie Michaelson, Nathan King, Karen A. Patte, Pauli Gardner, William Pickett

**Affiliations:** 1 Department of Health Sciences, Brock University, 1812 Sir Isaac Brock Way, St. Catharines, ON L2S 3A1 Canada; 2 Department of Public Health Sciences, Queen’s University, Kingston, ON Canada

**Keywords:** Health equity, Spiritual health, Determinants of health, Child and adolescent health, Epidemiology, Mental health, Spirituality

## Abstract

**Background:**

The health of adolescents is determined by structural and intermediary factors. Such factors operate through pathways that foster different opportunities to achieve health and wellbeing, contributing to inequities. Past analyses of cross-national adolescent health data show that measures of child spirituality, conceptualized as the strength of the connections in our lives, may operate as intermediary determinants in some Western countries. Inspired by this idea, the current analysis provides an in-depth exploration of such pathways among Canadian adolescents. Our objectives were to confirm the existence of relationships between economic position and seven indicators of adolescent health status, then explore whether any observed inequities could be explained by the strength of connections afforded by a healthy spirituality.

**Methods:**

Cycle 8 of the Canadian Health Behaviour in School-aged Children (HBSC) study was conducted in 2017–18. A school-based sample (*n* = 18,962) of adolescents was obtained from across Canada following a standard cross-national protocol. Eligible participants completed a general survey about their health, health behaviours and their determinants. Survey data were used to model the potential effect of perceived levels of relative affluence on each of seven health indicators. Comparison of crude and adjusted relative risks estimates from weighted log-binomial regression models provided evidence of indirect mediating effects attributable to each of four domains of spirituality.

**Results:**

As perceived levels of family affluence increased, the percentages of young people who reported each (7/7) of the negative health outcomes decreased. The spiritual health domain “connections to self” (i.e., *the importance of meaning, purpose, joy and happiness in life*) mediated the strength of relationships between relative affluence and each (7/7) of the outcomes in boys and girls. “Connections to others” (*the importance of kindness, respect and forgiveness*) mediated the strength of relationships between relative affluence and each (7/7) of the outcomes among girls. Inconsistent evidence of possible mediation was identified for connections to others in boys, as well as the other two domains of spirituality (*connections to nature,* then *connections to the transcendent*) in boys and girls.

**Conclusion:**

Specific connections afforded by a healthy spirituality could be intermediary determinants of health in Canadian adolescent populations.

**Supplementary Information:**

The online version contains supplementary material available at 10.1186/s12889-023-16060-5.

## Introduction

Childhood health experiences are shaped by the broader contexts of young peoples’ lives – the conditions in which they live, learn, grow, develop, and play [1]. Resources that positively shape these conditions, such as adequate household income, food security, and stable housing, are broadly understood as the “social determinants of health” [1]. The ways in which the distribution of such determinants relate to health inequities are summarized in a conceptual framework articulated by the World Health Organization’s *Commission on the Social Determinants of Health (“the Commission”)* [2]. This framework outlines how socioeconomic and political contexts shape socioeconomic positions, which in turn lead to the stratification of populations by factors such as income, education, occupation, gender and race [2]. Unequal distribution of the determinants of health privileges some while disadvantaging others, and typically leads to health inequities [3]. Evidence for such inequities includes gradients in health and health experiences observed across socio-economic and other strata of child and more general populations [1–4].

Beyond basic survival, having stable access to the social determinants of health empowers children and enables them to lead flourishing lives [4]. Mechanisms and pathways through which determinants operate at different stages of childhood are informative. The *Commission’s* framework refers to these as “intermediary determinants”, and they include material conditions that relate to affluence, psychosocial circumstances, and behavioural and/or biological factors [2]. Yet intermediary determinants also include “non-tangible resources” [4] that foster health in children’s lives and enable flourishing. When the quality of these “non-tangible resources” [4] is unequally distributed among people from different socioeconomic positions, health inequities emerge [4]. Children in such circumstances may not have the resources they need to achieve optimal health, nor the chance to make health promoting behavioural choices [4]. However, population-based studies of such “non-tangible resources” [4] as intermediary determinants of health are rare in the child health literature, and original studies of this concept may provide a novel evidence base in support of the development of potential interventions.

In past cross-national analyses of adolescent populations (Michaelson et al, Establishing spirituality as an intermediary determinant of health among 42,843 children from eight countries, In review). We have shown that measures of child spirituality, conceptualized as the strength of the connections in our lives [5, 6], may operate as intermediary determinants of health. While compelling, evidence in support of this idea was mixed, as the pathway was verified in Western but not Eastern European countries and involved models that were limited to a single health outcome (Michaelson et al, Establishing spirituality as an intermediary determinant of health among 42,843 children from eight countries, In review). Past analysis has also posited that spirituality is best conceptualized and analyzed in four relational domains (to self, to others, to nature and to the transcendent) [5–7]. When measured in this way, spirituality and its domains have been shown to be correlated to economic positions [8] and are also highly correlated with various health outcomes [9, 10]. It is therefore plausible that spirituality is in the pathway linking economic positions to health, operating alongside other primary mechanisms that are known intermediary determinants.” [2] New evidence focused on these pathways could support clinical and health promotion efforts that foster “human flourishing” [4], and address some of the intangible barriers that adolescents face in reaching their full potential.

The need for a more in-depth exploration of these potential pathways in specific contexts is warranted. We therefore conducted such an analysis with Canadian adolescents, with two objectives. *First,* we confirmed whether relationships between economic position and adolescent health were strong and consistent using a robust, contemporary sample of Canadian adolescents [11]. *Second,* we explored whether such relationships could be explained in part by the strength of connections afforded by a healthy spirituality, as implied by the Commission’s framework [2]. *A priori*, we examined both objectives by gender due to the substantially higher level of mental health problems reported by girls in Canada [12]. We examined a range of seven health outcomes to examine for consistent effects, in support of eventual causal inference. We also performed analyses by domain of spirituality, as influenced by past psychometric analyses [13]. Our goals were to broaden understanding of the novel ways that systemic inequities may arise in child populations, and to provide new etiological evidence to support the contention that spirituality is an unrecognized determinant of health, worthy of focused attention.

## Methods

### Data source

Health Behaviour in School-aged Children (HBSC) involves a cross-national general health survey administered every four years and involving children aged 11–15 years [14]. Canada has taken part in HBSC since 1989–90, and the 2017–18 survey represented its eight cycle in our country [11]. Recruitment in Canada involved a multi-stage sampling design, with participants nested within schools, then school boards, then provinces and Territories. Sampling was stratified by type of school and geographic regions on a replacement basis, whereby a non-participating school was replaced by a consenting school from the same board that had similar characteristics in terms of size of the student body, community size, language of instruction, and type of school board (public or separate). Participating students completed an anonymous questionnaire in a paper or online format during a -60- minute classroom session. Questionnaires were returned to Queen’s University for data entry, cleaning, and analysis.

### Ethical considerations

HBSC Canada received ethics approval from the federal government and our respective institutions. Informed consent was obtained from all participants and/or their legal guardian(s). Parental consent was active or passive, dependent upon local school board requirements, and child assent was also obtained. The study protocol met the institutions’ guidelines for protection of human subjects concerning their safety and privacy, and all methods were carried out in accordance with relevant guidelines and regulations.

### Key variables

#### Overview

Using the *Commission’s* framework [2] as an organizational structure, we tested potential etiological pathways that linked adolescent socio-economic positions with seven different health outcomes, and then explored whether the domains of spiritual health could potentially mediate such relationships, controlling for key covariates. We were limited to available measures from the 2017–18 Canadian HBSC, as this involved a secondary analysis of existing health survey data. A brief description of the measures used in this study is provided below. For a full description please see Supplementary Table 1.

#### Socio-economic positions (exposure)

Each HBSC participant described their *relative socio-economic position.* measured using a single item describing “how well off is your family”, with five categories ranging from “very well off” to “not at all well off” [11]. They also answered the following six items in the Family Affluence Scale (FAS III) describing material conditions in the household: “*Do you have your own bedroom for yourself?*”, “*How many bathrooms (room with a bath/shower or both) are in your home?*”, “*Does your family own a car, van or truck?*”, “*How many times did you and your family travel out of Canada for a holiday/vacation last year?*”, “*Does your family have a dishwasher at home?*”, and “*How many computers does your family own?*” [15]. Scores on the composite scale range from 0 to 13, and students were categorized using the lowest 20%, middle 60%, and highest 20% of the sample distribution (0 to 5 – “low affluence”; 6–10 – “medium affluence”; 11–13 – “high affluence)” [16].

#### Spiritual health (mediator)

Participants answered 10 questions in four domains with response options following a 5-point scale ranging from 0- “not at all important” to 4- “very important” [13]. Individual items described how important it was for them to: “feel that your life has meaning or purpose”; “experience joy (pleasure, happiness) in life” (the “connections to self” domain); “be kind to other people”; “be forgiving of others”; “show respect for other people” (connections to others); “feel connected to nature”; “care for the natural environment” (connections to nature); and “meditate or pray”; “feel a connection to a higher spiritual power”; “feel a sense of belonging to something greater than yourself” (connections to the transcendent). Summary scores were obtained for each domain. Cronbach alpha coefficients describing the internal consistencies of the overall scale and sub-scales in the four domains were acceptable (all > 0.7), and confirmatory factor analysis shows that these items fit the underlying latent constructs being summarized and that the module was best analyzed by domain [13].

#### Poor mental health (outcomes)

Seven different health outcomes measured using standard indicators were examined. For each, we dichotomized outcomes in order to maximize cell sizes and to adhere to established precedents surrounding their categorization [11, 14]. The outcomes were: (1) *fair or poor self-rated health*, based on a single item where health status was rated from ‘excellent” to “poor” [17]; (2) *low life satisfaction*, based on a score of 0–5 on the 10-point Cantril ladder [18], where 0 is a rating of the “worst possible life” and 10 is a rating of “the best possible life”; (3) *low resilience*, based on a low score (0–3 out of 8) on a 2-item scale where participants rated their ability to handle… “unexpected and difficult problems?” and “day-to-day demands in your life” [11]; (4) *frequent health complaints,* based on simultaneously experiencing at least two of eight subjective health symptoms [19] “more than once a week” or “About every day” during the past 6 months; (5) *low wellbeing,* based on a score of 50 or less on the WHO-5 [20] index; (6) *high emotional problems,* based on an average response of “agree” or more to the 5-item HBSC emotional problems scale [14]; and (7) *hopelessness, i.e., feeling sad or hopeless* (“yes “ vs. “no”), experienced almost every day over a two week period during the past 12 months [21].

#### Potential covariates

Students reported the date of survey completion and their month and year of birth, from which age was estimated. They reported whether they were a “boy” or “girl” (gender diverse responses were suppressed due to ethics restrictions involving small cell sizes). Their geographic region of origin was categorized by province or Territory (*West* – British Columbia, Alberta, Saskatchewan, Manitoba; *Central* – Ontario, Quebec; *East* – New Brunswick, Nova Scotia, Prince Edward Island, Newfoundland and Labrador; *North* – Yukon and Northwest Territories). Immigration status was categorized as “born in Canada”, or lived in Canada for “more than 5 years”; or “5 years or less” [22]. Ethnicity was categorized using Statistics Canada designations [23], later grouped into seven categories based on precedent [24].

### Statistical analysis

Analyses were conducted in SAS Version 9.4 (SAS Institute, Cary, NC). We restricted the sample to students with complete data on key variables of interest. A weighting variable was applied to ensure national representativeness by grade and geographic region, and all analyses were stratified by gender. The study sample was profiled demographically. We categorized the participants into three groups according to their perceived levels of affluence, and then estimated the percentage of young people reporting experiences of 7 negative health outcomes using descriptive (cross-tabulations) then analytic (weighted log-binomial regression) methods that controlled for age, clustering by school, and incorporated survey weights. Controlling for additional covariates (geographic region, immigration status, and ethnicity) did not impact the effect of affluence on the mental health outcomes, or the mediation effects of spiritual health (changes in effect estimates were < 5% across all models). Given that this additional adjustment did not impact the findings and to maximize the available sample these additional covariates were not controlled for in the regression models. Generalized Estimating Equations (GEEs) were used to adjust for clustering by school [25]. We next modelled the potential effect of perceived levels of relative affluence on each of the 7 mental health indicators in boys and girls, with and without adjustment for the four spiritual health domains (28 models total). Shifts in the relative risk for the outcomes towards the null provided evidence of a potential indirect mediating effect of the spiritual health domain on the relationship between socio-economic position and mental health. Based on final regression models, we also calculated the percentage of the total effect that was accounted for by each of the spiritual health domains; an approach referred to as the Difference Method [26].

## Results

Participants are profiled in Table 1 by available socio-demographic factors. The weighted sample consisted of 8,877 boys (mean age 13.8 years) and 10,085 girls (13.9 years); 301 participants (weighted n) who described themselves as having another gender identity were excluded, to adhere to ethics requirement. Most (95%) of the full sample were either born in Canada or lived in Canada for more than five years. The ethnic composition of the sample was reflective of the diversity of Canada [23]. The sample included a range of young people from varying socio-economic positions.

**Table 1:** Description of the sample, by gender

	**Boys** (*n* = 8,877)		**Girls** (*n* = 10,085)	
	n	(%)	n	(%)
Age, *Mean(SD)*	*13.8 (1.4)*		*13.9 (1.4)*	
*(Range)*	*(9.9 – 18.8)*		*(9.2 – 18.3)*	
≤ 11	994	(11.2)	1099	(10.9)
12	1830	(20.6)	1903	(18.9)
13	1825	(20.6)	2163	(21.5)
14	1954	(22.0)	2247	(22.3)
≥ 15	2275	(25.6)	2672	(26.5)
Geography				
West	2955	(33.3)	3202	(31.8)
North	43	(0.5)	42	(0.4)
Central	5262	(59.3)	6189	(61.4)
East	619	(7.0)	652	(6.5)
Immigration Status				
Born in Canada	6042	(72.4)	7305	(77.5)
Lived in Canada > 5 years	1872	(22.5)	1616	(17.1)
Lived in Canada ≤ 5 years	433	(5.2)	504	(5.4)
*Missing*	*532*		*659*	
Ethnicity				
White	6287	(71.7)	6965	(70.1)
Indigenous	264	(3.0)	307	(3.1)
Black	334	(3.8)	452	(4.6)
East & Southeast Asian	291	(3.3)	293	(3.0)
East Indian & South Asian	299	(3.4)	365	(3.7)
Arab & West Asian	155	(1.8)	184	(1.9)
Other (including mixed)	1142	(13.0)	1370	(13.8)
*Missing*	*108*		*149*	
Family Affluence Scale (0–13)				
Low (0–5)	1363	(15.9)	1898	(19.2)
Medium (6–10)	5176	(60.3)	5730	(58.0)
High (11–13)	2040	(23.8)	2260	(22.9)
*Missing*	*300*		*196*	
Relative Family Affluence				
Not welloff	631	(7.3)	856	(8.8)
Average	2901	(33.7)	3683	(38.0)
Welloff	5064	(58.9)	5162	(53.2)
*Missing*	*282*		*384*	

(1) All values are weighted, (2) due to ethics (privacy) requirements, 301 students who identified as having another gender identity were excluded

Table 2 describes the percentages of boys and girls reporting each of seven health-related outcomes (in dichotomous format) according to their perceived level of socio-economic status, as indicated by the relative affluence measure (N.B., for completeness, these outcomes are described according to their full range of response options in Supplementary Tables 2A (boys) and 2B (girls). Two main findings were evident. *First,* among both boys and girls, as the level of relative family affluence increased, the percentages of young people who reported each of the seven negative health outcomes decreased (all p_trend_ < 0.001). *Second,* irrespective of their level of relative family affluence, girls consistently reported higher levels of the negative health outcomes than did boys (all *p* < 0.001). (N*.B., findings using the FAS III scale were similar, are available upon request, and are not reported here for reasons of parsimony*).

**Table 2:** Percentages of young people reporting sentinel indicators of poor mental health by relative family affluence, boys and girls, Canada, 2018

	**Boys**				**Girls**			
	**n Total**	**Not welloff**	**Average**	**Welloff**	**n Total**	**Not welloff**	**Average**	**Welloff**
**Outcomes:**		**%Yes**	**%Yes**	**%Yes**		**%Yes**	**%Yes**	**%Yes**
Self-Rated Health, *Fair or Poor*	8551	26	18	11	9627	32	24	12
Life Satisfaction, *Low*	8464	27	16	8	9578	41	28	14
Resilience, *Low*	8460	22	19	12	9490	37	29	20
Health Complaints, *Frequent*	8207	39	28	19	9304	60	48	38
Well-being,* Low*	8371	32	24	12	9484	50	38	22
Emotional Problems, *High*	8314	17	9	6	9363	36	21	13
Hopelessness, *Yes*	8395	38	27	17	9493	54	42	31

(1) All values are weighted to ensure that each province/Territory is proportionally represented by grade, (2) The following categories were used to describe the negative mental health outcomes: ‘Fair’ or ‘Poor’ vs ‘Good’ or Excellent’ self-rated health, Low life satisfaction = 0–5 out of 10 on the Cantril Ladder, Low resilience = 0–3 out of 8, Frequent health complaints = experiencing at least 2 of 8 health complaints at least weekly, low well-being = 50 or less out of 100 on the WHO-5 Well-being Index, high emotional problems = 15 or higher out of 20 on the 5-item HBSC Emotional Problems scale, and Feelings of hopelessness (yes or no) was indicated if students reported feeling so sad or hopeless almost every day for two weeks or more in a row that they stopped doing some usual activities

We further explored the relationships between perceived socio-economic position and each of the seven study outcomes. While the prevalence of each outcome was higher in girls than boys (Table 2), relationships between socio-economic position and health were consistent (Table 3). As perceptions of socio-economic position increased, reports of negative health outcomes decreased in both genders examined. Among girls, those from relatively welloff families were 66% less likely to report low life satisfaction (RR: 0.34; 95% CI: 0.29–0.39; *p* < 0.001)) and 41% less likely to reporting feelings of hopelessness (RR: 0.59; 95% CI: 0.53–0.66; *p* < 0.001) than those from not welloff families. Similarly, boys from welloff families were 70% less likely to report low life satisfaction (RR: 0.30; 95% CI: 0.24–0.37; *p* < 0.001)), and 54% less likely to report feelings of hopelessness (RR: 0.46; 95% CI: 0.39–0.55; *p* < 0.001).

**Table 3:** Associations between level of self-perceived family affluence and seven sentinel negative mental health outcomes, by gender. A Relative Risk (RR) < 1.00 suggests that higher family affluence is associated with a lower likelihood of negative mental health outcomes

	**Boys**					**Girls**				
	**Not welloff**	**Average**		**Welloff**		**Not welloff**	**Average**		**Welloff**	
**Negative Outcomes:**	RR	RR	(95% CI)	RR	(95% CI)	RR	RR	(95% CI)	RR	(95% CI)
Self-Rated Health, *Fair or Poor*	1.00	0.69	(0.56–0.85)	0.43	(0.36–0.52)	1.00	0.73	(0.63–0.85)	0.39	(0.33–0.46)
Life Satisfaction, *Low*	1.00	0.60	(0.48–0.74)	0.30	(0.24–0.37)	1.00	0.67	(0.58–0.78)	0.34	(0.29–0.39)
Resilience, *Low*	1.00	0.86	(0.65–1.14)	0.55	(0.42–0.73)	1.00	0.77	(0.67–0.89)	0.51	(0.43–0.60)
Health Complaints, *Frequent*	1.00	0.72	(0.61–0.85)	0.51	(0.43–0.60)	1.00	0.77	(0.70–0.85)	0.63	(0.57–0.69)
Well-being,* Low*	1.00	0.72	(0.59–0.88)	0.37	(0.30–0.46)	1.00	0.72	(0.65–0.80)	0.44	(0.39–0.49)
Emotional Problems, *High*	1.00	0.53	(0.39–0.73)	0.33	(0.24–0.44)	1.00	0.55	(0.47–0.65)	0.37	(0.31–0.43)
Hopelessness, *Yes*	1.00	0.70	(0.60–0.81)	0.46	(0.39–0.55)	1.00	0.76	(0.67–0.86)	0.59	(0.53–0.66)

RR’s adjusted for age and clustering by school, and weighted to ensure that each province/Territory is proportionally represented by grade

**Fig. 1:**
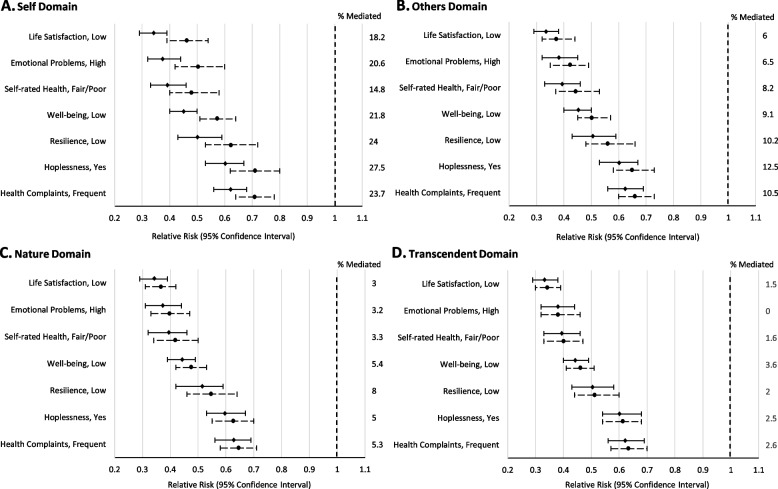
The proportions of the protective effect of high (vs low) relative family affluence on 7 indicators of poor mental health mediated by connections to: oneself (Panel **A**), others (Panel **B**), nature (Panel **C**), and the transcendent (Panel **D**) in girls. Each panel displays the relative risk and 95% confidence interval for each outcome before (—) and after (-----) adjusting for the domain of spiritual health. Note that all models are adjusted for age and clustering by school, and sampling weights were applied to ensure that each province/Territory was proportionally represented by grade

**Fig. 2:**
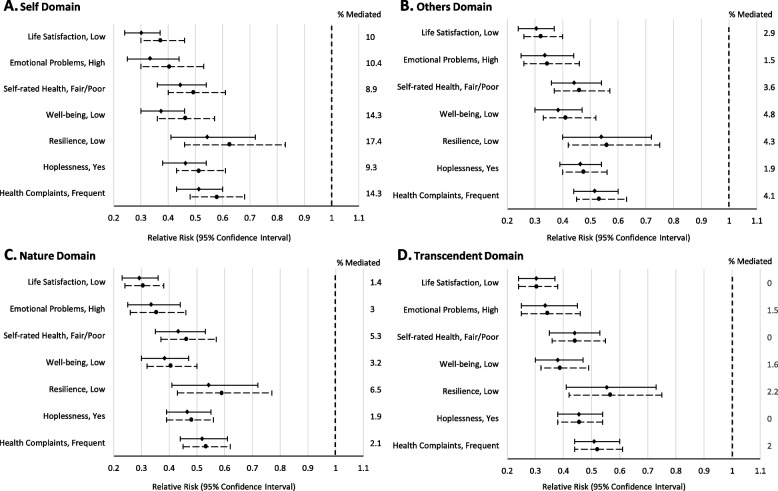
The proportions of the protective effect of high (vs low) relative family affluence on 7 indicators of poor mental health mediated by connections to: oneself (Panel **A**), others (Panel **B**), nature (Panel **C**), and the transcendent (Panel **D**) in boys. Each panel displays the relative risk and 95% confidence interval for each outcome before (—) and after (-----) adjusting for the domain of spiritual health. Note that all models are adjusted for age and clustering by school, and sampling weights were applied to ensure that each province/Territory was proportionally represented by grade

We next examined the potential mediating effects of the four spiritual health domains. Among girls (Fig. 1), when the “connections to self” measure was included in our models, the protective effect of higher family affluence on risks for the outcomes was reduced. Percentages of any direct effects that were mediated ranged from 14.8 to 27.5%. This pattern was also observed when including the measure for the domain “connections to others”, although the percentages of direct effects that were mediated were smaller (6.0 to 12.5%). Little potential mediation was observed for the other two domains (“connections to nature”; “connections to the transcendent”). Figure 2 presents a similar analysis conducted for boys. While the extent of the mediated effects was modest, the pattern was the same as for girls. Hence, connections in some, but not all, spiritual health domains appeared to act as intermediary determinants in social determinants of health models in both genders examined.

## Discussion

This unique analysis of Canadian adolescent health survey data had three main outcomes. *First,* we observed a clear social gradient in the reporting of all indicators of mental health status, with adolescents from more affluent backgrounds experiencing better health status. *Second,* we affirmed from these findings that girls reported experiencing lower levels of mental health outcomes compared with boys. And *third* and most central to our core objectives, we provide new evidence to support the idea that, consistent with social determinants of health theory [1–3], some aspects of spirituality (connections to “self” and “others”) may act as intermediary determinants in pathways that connect socio-economic positions to mental health status in populations of adolescents in Canada. For other domains (connections to “nature” and “the transcendent”), we found no evidence of such effects.

Our confirmation of a social gradient in the mental health experiences of children was expected, as it reaffirmed that affluence is correlated with positive aspects of health and wellbeing [1, 2]. Yet, even with this expectation the robustness of this pattern was striking. For each of the seven mental health indicators, a strong correlation was reported between higher affluence and lower levels of  poor mental health. This was true for both boys and girls. While the cross-sectional nature of our study precludes inference of causation, this finding is consistent with a strong body of evidence demonstrating that family affluence operates as a structural factor that directly affects the mental health status of children. The dose–response nature of the observed social gradients is also informative. This not only affirmed that Canadian children living with affluence are much less likely to experience mental health problems than those living in poverty, but that the observed social gradient applies to all young people across the full spectrum of affluence levels. To illustrate, children in the middle of the social hierarchy experience worse health than those who are more affluent, and better health than those who are poor. Hence, each rung of the metaphorical “social ladder” [27] relates to better (or worse) health than the rung before, even when the children on the middle rung are not living in poverty.

Observed differences in mental health status reported by girls *vs.* boys are also informative. Consistent with recent reports [11, 12, 14], adolescent girls in Canada report experiencing negative mental health outcomes much more frequently than do boys. This may be a real phenomenon, although some have attributed this pattern to gendered differences in the willingness to disclose mental health problems [28, 29]. Irrespective of its cause, the observed gendered differences were consistent across each of the seven self-reported mental health status indicators and were also evident within each of the three levels of affluence under study. It was also notable that once gendered differences in the prevalence of outcomes were controlled for via modeling, that the observed social gradient remained strong and consistent, irrespective of gender. This provides further evidence not only that affluence is a direct, underlying determinant of mental health status in young Canadians, but that a salient gap between the mental health of boys and girls exists. This is disconcerting. Canada prides itself on being a country that promotes gender equality [30] and this would be expected to translate to fewer differences in health experienced between boys and girls. However, recent cross-national analyses involving 73 countries [31] counter that hypothesis, demonstrating that as gender equality increases across countries so do differences in the prevalence of four salient mental health outcomes that disadvantage girls. Speculatively, this was attributed to the fact that recent advances in gender equity have caused women and girls to face a “double burden” of increased responsibility for leadership on top of traditional expectations in terms of female responsibilities and norms [31]. This may be especially complex for girls and young women in their formative development years. Deeper scrutiny of the complexity of gendered disparities in mental health outcomes is clearly warranted for Canada.

Findings from our modeling examining the four domains of spirituality as potential intermediary determinants of health are similarly provocative. Using the level of partial mediation as a guide, it appears that “connections to self” and, to a lesser extent, “connections to others”, may represent middle steps in etiological pathways that link material resources with mental health outcomes in children. This is not the first time that “connections to self and others” have been shown to be central in fostering health among populations of children [9], and our models confirm this finding for young people in Canada. Connections to self are driven by an internalized sense of both “meaning and purpose”, consistent with theories of salutogenesis [32]. The like finding for “happiness” as a potential mediator also resonates with theories of positive youth development [33]. Connections to others focus on experiences of respect, forgiveness, and kindness to others [5–9]. Hence, if young people experience deprivation, perhaps this can manifest itself in having fewer material resources available to live optimally, as well as the freedom to make choices to live meaningful lives. Perhaps deprivation robs children of the opportunities to have meaning and purpose, happiness, and deeper connections with others in their lives, and this in turn has negative health effects. This places barriers both internally and externally within the lives of children and sets up the potential for a trajectory towards negative mental health outcomes. This evidence is in keeping with our initial hypothesis that some aspects of spirituality act as intermediary determinants of adolescent health.

In two bodies of literature, a great deal has been written about the importance of both connections with nature [34, 35] and connections with the transcendent [36–38] as possible mechanisms through which specific populations of children achieve optimal health status. The latter is especially true in studies examining the role of institutionalized religion [36–38] as a protective health asset in the lives of young people, particularly around externalizing behaviours [39]. The transcendent domain examined in our analysis, while not focused on formal religion, does include indicators of the perceived importance of various religious belief and practices [6–10]. Hence, we were truly surprised to see how modest the indirect effects were for these potential intermediary determinants. The limited extent of mediation may in part be explained by the relatively low prevalence of young Canadians who rated the items in these particular domains as important [5, 10], lack of specificity in the items used to assess each domain’s importance, or the lack of a true effect. Alternatively, it is possible that connections to nature and the transcendent relate to health status only in very specific populations that value such experiences, or through more complicated pathways that consider variables not included in our models.

Limitations of our analysis warrant comment. Our use of cross-sectional data to study potentially causal pathways, means any relationships should be viewed as “correlations” and “potential effects”, and the identified pathways require confirmation via longitudinal analyses. We were also limited to available measures that are brief and targeted to meet cross-national survey requirements and the literacy levels of children as young as 11 years of age [11, 14]. Data from non-binary gender identifying children were not available due to small sample sizes and mitigation of the risk of reidentification. On the other hand, the strengths of this analysis include the robust nature of our sample, which is nationally representative and includes young people at formative ages from across our country. Additionally, measures used in our analysis have strong histories in terms of validity and reliability in child populations e.g., [13, 15, 17–20], and historical use in our 50 + country international research network e.g., [13–15, 19, 22, 24]. Finally, our statistical analysis was framed using an established theoretical framework [1, 2], and our core hypothesis was novel and builds on a strong base of theory and an initial cross-sectional analysis (Michaelson et al, Establishing spirituality as an intermediary determinant of health among 42,843 children from eight countries, In review).

Study findings provide targeted insight for future research priorities and prevention approaches. Some core findings confirm past observation and strengthen bodies of evidence on the effects of socio-economic disadvantage on the health of children [3–5], the potential effects of gender on mental health experiences [11, 17], and the connections between a healthy spirituality and positive health outcomes during adolescence [8–10]. Findings affirm and offer a reminder that these relationships, and the deep etiological reasons for their existence, remain important research priorities in Canada. These priorities are worthy of further investigation, both epidemiological and qualitative, in general and at-risk populations. Findings emergent from our examination of spirituality as an intermediary determinant are helpful in moving this field forward. As Marmot [4, 5] and Sen [40] have emphasized, much of the existing evidence to describe the social determinants of health focuses on the ‘causes of the causes’ of ill health. They argue that it is critical for our child research efforts to focus on the conditions that “empower people” and enable “flourishing lives” [4, 40]. To that end, our findings clearly show that promoting “meaning”, “purpose” and “happiness” in the lives of children, associated with the spirituality domain of “connections to self”, show great promise as unrecognized intermediary determinants of health. This is closely followed by relational experiences that foster “respect”, “forgiveness” and “kindness” to others. As inequities rise and poor health outcomes among lower income populations of children continue there is a clear and moral obligation to develop, test and implement policies and practices that ensure adequate resources are provided to adolescents that go beyond what is needed for basic survival and that enable flourishing. This must include attention to both material and non-material resources—such as spirituality. Our findings add to evidence that it is critical we redistribute basic material resources to create a more just society. Importantly our work also highlights the need to study, evaluate and implement new interventions that prioritize a focus on fostering opportunities to develop deep inner meaning within the child. The evidence of this research justifies investing in these aspects of spirituality as intermediary determinants of health. Further, it  represents an important contribution to the literature and, perhaps a new way forward in supporting a just society for all.

## Supplementary Information

**Additional file 1: Supplementary Table 1. **Description of study measures. **Supplementary Table 2A. **Description of mental health outcomes by relative family affluence in boys. **Supplementary Table 2B. **Description of mental health outcomes by relative family affluence in girls.

## Data Availability

The datasets on which the current study is based are not publicly available, but are available from the senior author and the HBSC International Coordinating Centre on reasonable request.
