# Supplementary material for: Connections associated with a healthy spirituality: are these unrecognized intermediary determinants that shape health inequities in Canadian young people?
Source: BMC Public Health. 2023 Jun 17;23:1167. doi: 10.1186/s12889-023-16060-5 (PMC10276412; doi:10.1186/s12889-023-16060-5)
Supplement: Supplementary file 1 — Additional file 1: Supplementary Table 1. Description of study measures. Supplementary Table 2A. Description of mental health outcomes by relative family affluence in boys. Supplementary Table 2B. Description of mental health outcomes by relative family affluence in girls. [file 12889_2023_16060_MOESM1_ESM.docx]

| **Supplementary Table 1.** Description of study measures | | | |
| --- | --- | --- | --- |
| **Outcomes:**  **Mental Health** | **Measure** | **Response Options** | **Coding** |
| Self-Rated Health | Would you say your health is…? | 1=Excellent, 2=Good, 3=Fair, 4=Poor | Fair or Poor *versus*  Good or Excellent |
| Hopelessness | During the last 12 months, did you ever feel so sad or hopeless almost every day for two weeks or more in a row that you stopped doing some usual activities? | 1=Yes, 2=No | Yes *vs.* No |
| Life Satisfaction  [Cantril Ladder] | In general, where on the ladder do you feel you stand at the moment? Mark the box next to the number that best describes where you stand. | 10= Best possible life to 0= Worst possible life | Low life satisfaction= 0-5 out of 10 |
| Resilience | How would you rate your ability to handle…   1. unexpected and difficult problems? 2. day-to-day demands in your life? | 0= Poor, 1= Fair, 2= Good, 3= Very Good, 4= Excellent | Low resilience= 0-3 out of 8 (average of less than good) |
| Health Complaints  [8-item HBSC Psychosomatic Complaints Scale] | In the last 6 months, how often have you had the following?  a. Headache  b. Stomachache  c. Backache  d. Feeling low (depressed)  e. irritability or bad temper  f. feeling nervous  g. difficulties in getting to sleep  h. feeling dizzy | 1= About every day  2= More than once a week  3= About every week  4= About every month  5= Rarely or never | Frequent health complaints= experiencing at least 2 of 8 symptoms more than once a week or about every day |
| Well-being  [WHO-5 Well-Being Index] | Over the last two weeks, how often have you…   1. felt cheerful and in good spirits? 2. felt calm and relaxed? 3. felt active and energetic? 4. woken up feeling fresh and rested? 5. had your daily life filled with things that interest you? | 5= All of the time  4= Most of the time  3= More than half the time  2= Less than half the time  1=Some of the time  0= At no time | Sum the 5 items and multiply by 5 so raw score= 0 to 100. Low well-being= 50 or less out of 100 |
| Emotional Problems  [5-item HBSC Emotional Problems Scale] | Please show how much you agree or disagree with the following statements.   1. I often wish I were someone else 2. I often feel helpless 3. I would change how I look if I could 4. I often feel left out of things 5. I often feel lonely | 4= Strongly agree  3=Agree  2= Neither agree nor disagree  1=Disagree  0=Strongly disagree | High emotional problems= average response of agree or strongly agree on the 5 items (≥15 out of 20) |
| **Exposure:**  **Family Affluence** |  |  |  |
| Relative Family Affluence | How well off do you think your family is? | 1= Very well-off  2= Quite well-off  3= Average  4= Not very well-off  5= Not at all well-off | Well-off= 1, 2  Average= 3  Not well-off= 4, 5 |
| Family Affluence | 1. Do you have your own bedroom for yourself? | 0= No, 1=Yes | High= top 20% of distribution (11-13)  Medium= middle 60% (6-10)  Low= bottom 20% (0-5) |
| [6-item FAS-III Scale] | 1. How many bathrooms (room with a bath/shower or both) are in your home? | 0= None, 1= One, 2= Two, 3= More than two |  |
|  | 1. Does your family own a car, van or truck? | 0= No, 1= Yes one, 2= Yes two or more |  |
|  | 1. How many times did you and your family travel out of Canada for a holiday/vacation last year? | 0= Not at all, 1= Once, 2= Twice, 3= More than twice |  |
|  | 1. Does your family have a dishwasher at home? | 0= No or 1= Yes |  |
|  | 1. How many computers does your family own? | 0= None, 1= Once, 2= Two, 3= More than two |  |
| **Mediators:**  **Spiritual Health Domains** | |  |  |
| ***Domains:*** | How important is it for you to… ? | 0= Not at all important to 4= Very important |  |
| Connections to Self | 1. Feel that your life has meaning or purpose. 2. Experience joy in life. |  | Sum of 2 items= 0-8 |
| Connections to Others | 1. Be kind to other people. 2. Be forgiving of others. 3. Show respect for other people. |  | Sum of 3 items= 0-12 |
| Connections to Nature | 1. Feel connected to nature or wilderness. 2. Care for the natural world. |  | Sum of 2 items= 0-8 |
| Connections to the Transcendent | 1. Meditate or pray. 2. Feel a connection to a higher spiritual power. 3. Feel a sense of belonging to something greater than yourself. |  | Sum of 3 items= 0-12 |
| **Stratifying Variable (Effect Modifier):** | |  |  |
| Gender | Are you male or female? | Male  Female  Neither term describes me | Boy or Girl (gender diverse responses supressed due to ethics restrictions) |
| **Potential Covariates:** |  |  |  |
| Age | Age in years calculated from date of survey completion and month and year of birth | Age in years | Age in years |
| Geographic Region | Province or Territory where the student’s school is located | Province or Territory | West= BC, Alberta, Saskatchewan, Manitoba  East= New Brunswick, Nova Scotia, PEI, Newfoundland & Labrador  Central= Ontario, Quebec  North= Yukon, NWT |
| Immigration Status | How many years have you lived in Canada? | 1= I was born in Canada  2= 1 to 2 years  3= 3 to 5 years  4= 6 to 10 years  5= 11 or more years | Collapsed into 3 categories: Born in Canada, Lived in Canada >5 years, Lived in Canada ≤5 years |
| Ethnicity | People living in Canada come from many different cultural and racial backgrounds. How do you describe yourself? | Response Options: White, Chinese, South Asian, Black, Filipino, Latin American, Southeast Asian, Arab, Metis, Inuit, First Nations, Japanese, Korean, West Asian, or Other (please specify) | Grouped into 7 categories: White, Indigenous, Black, East & Southeast Asian, East Indian & South Asian, Arab & West Asian, or Other (including mixed) |

| **Supplementary Table 2A.** Description of mental health outcomes by relative family affluence in boys | | | | | | | |
| --- | --- | --- | --- | --- | --- | --- | --- |
|  | | **Not well-off** | | **Average** | | **Well-off** | |
| Self-Rated Health | | **n** | **(%)** | **n** | **(%)** | **n** | **(%)** |
|  | Excellent | 182 | (24.7) | 705 | (22.7) | 1759 | (36.4) |
|  | Good | 375 | (50.9) | 1770 | (56.9) | 2526 | (52.3) |
|  | Fair | 152 | (20.6) | 574 | (18.5) | 489 | (10.1) |
|  | Poor | 28 | (3.8) | 62 | (2.0) | 54 | (1.1) |
| Hopelessness | |  |  |  |  |  |  |
|  | Yes | 258 | (35.6) | 807 | (26.7) | 876 | (18.7) |
|  | No | 467 | (64.4) | 2221 | (73.4) | 3817 | (81.3) |
| Life Satisfaction | |  |  |  |  |  |  |
|  | Low (0-5) | 198 | (27.2) | 541 | (17.6) | 427 | (8.9) |
|  | Moderate (6-8) | 346 | (47.5) | 1875 | (61.1) | 2511 | (52.6) |
|  | High (9-10) | 184 | (25.3) | 654 | (21.3) | 1836 | (38.5) |
| Resilience | |  |  |  |  |  |  |
|  | Low (0-3) | 166 | (22.9) | 624 | (20.2) | 582 | (12.2) |
|  | Moderate/High (4-8) | 560 | (77.1) | 2464 | (79.8) | 4173 | (87.8) |
| Health Complaints | |  |  |  |  |  |  |
|  | Frequent (≥2 symptoms at least weekly) | 258 | (37.1) | 810 | (27.4) | 969 | (20.8) |
|  | Infrequent | 437 | (62.9) | 2151 | (72.6) | 3681 | (79.2) |
| Well-being | |  |  |  |  |  |  |
|  | Low (≤50) | 209 | (29.1) | 709 | (23.2) | 611 | (13.0) |
|  | Moderate/High (51-100) | 509 | (70.9) | 2348 | (76.8) | 4095 | (87.0) |
| Emotional Problems | |  |  |  |  |  |  |
|  | High (15-20) | 123 | (17.2) | 298 | (9.8) | 269 | (5.7) |
|  | Moderate/Low (0-14) | 594 | (82.9) | 2756 | (90.2) | 4418 | (94.3) |
|  |  | **Mean** | **(SD)** | **Mean** | **(SD)** | **Mean** | **(SD)** |
| Life Satisfaction (0-10) | | 6.8 | (2.4) | 7.1 | (1.8) | 7.9 | (1.7) |
| Resilience (0-8) | | 4.7 | (1.9) | 4.8 | (1.7) | 5.3 | (1.7) |
| Well-being (0-100) | | 62.9 | (24.3) | 63.9 | (20.1) | 71.1 | (19.1) |
| Health Complaints, at least weekly (0-8) | | 1.49 | (1.94) | 1.10 | (1.57) | 0.84 | (1.44) |
| Emotional Problems (0-20) | | 8.3 | (5.6) | 7.4 | (5.0) | 5.9 | (4.8) |
|  | | **Median** | **(IQR)** | **Median** | **(IQR)** | **Median** | **(IQR)** |
| Health Complaints, at least weekly (0-8) | | 1 | (0-2) | 0 | (0-2) | 0 | (0-1) |
| Note: SD= Standard Deviation, IQR= Interquartile Range | | | | | |  |  |

| **Supplementary Table 2B.** Description of mental health outcomes by relative family affluence in girls | | | | | | | |
| --- | --- | --- | --- | --- | --- | --- | --- |
|  | | **Not well-off** | | **Average** | | **Well-off** | |
| Self-Rated Health | | **n** | **(%)** | **n** | **(%)** | **n** | **(%)** |
|  | Excellent | 155 | (18.0) | 609 | (16.3) | 1528 | (31.5) |
|  | Good | 413 | (48.0) | 2147 | (57.5) | 2681 | (55.3) |
|  | Fair | 231 | (26.8) | 878 | (23.5) | 567 | (11.7) |
|  | Poor | 62 | (7.2) | 98 | (2.6) | 69 | (1.4) |
| Hopelessness | |  |  |  |  |  |  |
|  | Yes | 490 | (57.9) | 1723 | (46.9) | 1563 | (32.8) |
|  | No | 356 | (42.1) | 1953 | (53.1) | 3203 | (67.2) |
| Life Satisfaction | |  |  |  |  |  |  |
|  | Low (0-5) | 387 | (45.3) | 1140 | (30.7) | 749 | (15.6) |
|  | Moderate (6-8) | 328 | (38.4) | 2020 | (54.5) | 2583 | (53.7) |
|  | High (9-10) | 139 | (16.3) | 549 | (14.8) | 1480 | (30.8) |
| Resilience | |  |  |  |  |  |  |
|  | Low (0-3) | 314 | (37.1) | 1111 | (30.1) | 865 | (18.2) |
|  | Moderate/High (4-8) | 532 | (62.9) | 2586 | (70.0) | 3902 | (81.9) |
| Health Complaints | |  |  |  |  |  |  |
|  | Frequent (≥2 symptoms at least weekly) | 497 | (61.3) | 1804 | (50.2) | 1786 | (38.3) |
|  | Infrequent | 314 | (38.7) | 1788 | (49.8) | 2880 | (61.7) |
| Well-being | |  |  |  |  |  |  |
|  | Low (≤50) | 422 | (50.3) | 1447 | (39.5) | 1088 | (22.9) |
|  | Moderate/High (51-100) | 417 | (49.7) | 2214 | (60.5) | 3667 | (77.1) |
| Emotional Problems | |  |  |  |  |  |  |
|  | High (15-20) | 306 | (36.6) | 879 | (24.0) | 691 | (14.8) |
|  | Moderate/Low (0-14) | 530 | (63.4) | 2791 | (76.1) | 3995 | (85.3) |
|  |  | **Mean** | **(SD)** | **Mean** | **(SD)** | **Mean** | **(SD)** |
| Life Satisfaction (0-10) | | 5.9 | (2.4) | 6.5 | (2.0) | 7.4 | (1.9) |
| Resilience (0-8) | | 4.1 | (2.0) | 4.3 | (1.8) | 5.0 | (1.8) |
| Well-being (0-100) | | 51.1 | (25.6) | 55.3 | (21.5) | 64.7 | (20.9) |
| Health Complaints, at least weekly (0-8) | | 2.84 | (2.53) | 2.10 | (2.18) | 1.58 | (1.97) |
| Emotional Problems (0-20) | | 11.5 | (6.0) | 10.3 | (5.3) | 8.2 | (5.4) |
|  | | **Median** | **(IQR)** | **Median** | **(IQR)** | **Median** | **(IQR)** |
| Health Complaints, at least weekly (0-8) | | 2 | (0-5) | 2 | (0-3) | 1 | (0-3) |
| Note: SD= Standard Deviation, IQR= Interquartile Range | | | | | |  |  |
